# The multi-omics insights into mitochondrial dysfunction in the pathogenesis of cholelithiasis

**DOI:** 10.1097/MD.0000000000044525

**Published:** 2025-09-12

**Authors:** Haiyan Hou, Zhuyi Jiang, Liying Zhu

**Affiliations:** a Department of Infectious Diseases, The Second Affiliated Hospital of Harbin Medical University, Harbin Medical University, Harbin, Heilongjiang, China; b Department of Dermatology, China-Japan Friendship Hospital, Capital Medical University, Beijing, China.

**Keywords:** cholelithiasis, gene expression, methylation, mitochondria, summary-data-based Mendelian randomization

## Abstract

Cholelithiasis is the most prevalent biliary disease globally, and mitochondrial dysfunction has been implicated in its pathogenesis. However, the exact mechanisms remain poorly understood. In this study, we used Summary-data-based Mendelian randomization (SMR) and colocalization analysis, integrating multi-omics data, to investigate the association between mitochondrial-related genes and cholelithiasis. Summary-level quantitative trait loci (QTL) data at methylation, RNA, and protein levels were retrieved from European cohort studies. We integrated multi-omics data, including methylation QTL (mQTL), expression QTL (eQTL), and protein QTL (pQTL), alongside genome-wide association studies (GWAS) data from FinnGen and the UK Biobank. SMR and colocalization analysis were employed to evaluate the causal relationship between mitochondrial-related genes and cholelithiasis. Potential therapeutic targets for cholelithiasis were further validated through phenome-wide association studies (PheWAS), functional enrichment analysis, protein–protein interaction networks (PPI), drug prediction, and molecular docking. Following integration of the multi-omics evidence, we identified 4 mitochondrial-related genes, categorized by evidence strength as: Tier 1 genes (supported by 2 omics and colocalization evidence): *LIAS, HEBP1, PNKD*; Tier 2 genes (supported by 2 omics): *TARS2*. PheWAS analysis indicated that these 4 genes were not associated with other traits. Biologically, these genes are closely related to metabolic processes. Molecular docking analysis showed high binding affinities for candidate drugs, including olmesartan and neostigmine bromide. By integrating multi-omics data, we have constructed the first causal chain of linking mitochondrial-related genes, metabolic pathways, and cholelithiasis. This study provides a theoretical foundation for personalized therapies targeting the genes *LIAS, TARS2, HEBP1*, and *PNKD*.

## 1. Introduction

Cholelithiasis, also known as gallstones, is a condition characterized by the formation of calculi within the bile ducts or gallbladder. These stones may migrate and obstruct the biliary tract, leading to complications such as severe abdominal pain, jaundice, acute pancreatitis, or secondary infection. As the most common biliary disease worldwide, cholelithiasis represents a significant public health burden.^[1]^ Mitochondria are double-membrane-bound organelles found in most eukaryotic cells and are often referred to as the “powerhouses” of the cell. They play a crucial role in cellular energy production through oxidative phosphorylation (OXPHOS) and the generation of adenosine triphosphate (ATP), participating in the metabolism of glucose and lipids.^[2–4]^ Therefore, abnormalities in mitochondrial DNA (mtDNA) can affect ATP production, leading to common metabolic disorders such as type 2 diabetes, hypertension, and dyslipidemia.^[5–7]^ In recent years, research on the association between mitochondrial dysfunction and cholelithiasis has garnered significant attention. Although the crucial role of mitochondria in the pathogenesis of cholelithiasis is well established, specific mitochondrial-related genes and their downstream effects on cholelithiasis have yet to be fully elucidated.^[8,9]^

Mendelian randomization (MR) analysis uses genetic variants as instrumental variables to strengthen causal inferences between exposure and outcome. Compared to observational studies, this method is less susceptible to confounding variables and reverse causality effects, since genetic variation is randomly distributed during conception and is unaffected by disease onset. Summary-data-based Mendelian randomization (SMR) is applied in MR analyses related to gene expression. When exposure and outcome are derived from 2 independent large cohorts, SMR can achieve much higher statistical power than traditional MR analysis.^[10]^ The increasing availability of large-scale genome-wide association studies (GWAS) and quantitative trait locus (QTL) data has enabled us to explore the causal relationship between mitochondria-associated gene regulation and cholelithiasis, focusing on methylation, gene expression, and protein abundance levels. In this study, we utilized SMR analysis to examine the association of mitochondrial-related gene methylation, gene expression, and protein abundance levels with cholelithiasis risk.

## 2. Methods

**Figure 1.**
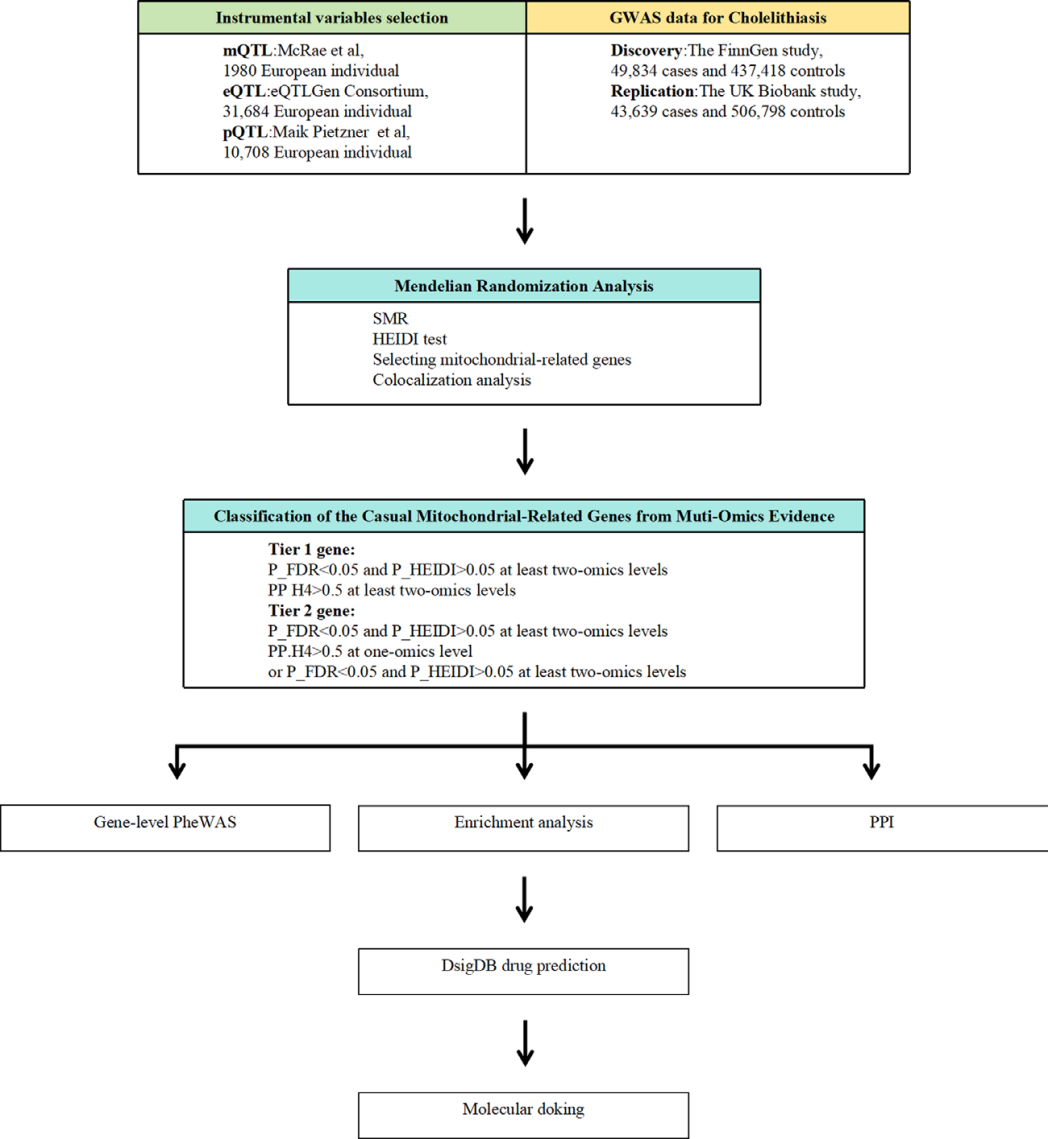
Study design. FDR = false discovery rate, HEIDI = Heterogeneity In Dependent Instrument, P_FDR = FDR-adjusted *P*-value, PP.H4 = posterior probability of H4, PPI = protein–protein interaction, QTL = quantitative trait loci, SMR = summary-based Mendelian randomization.

In this study, we utilized SMR analysis, incorporating the following publicly available genetic datasets: the FinnGen study (R12) as the discovery cohort and the UK Biobank as the validation cohort (Table S1, Supplemental Digital Content, https://links.lww.com/MD/P940). The FinnGen study (R12) dataset (https://www.finngen.fi/fi) served as the primary dataset for the main findings of this study. For independent validation, we used data from the UK Biobank.^[11]^ In this study, summary-level QTL data (including methylation QTL (mQTL), gene expression QTL (eQTL), and protein QTL (pQTL)) were used as instrumental variables to represent gene methylation, gene expression, and protein abundance levels. We used these QTL data for SMR analysis of cholelithiasis and applied the Heterogeneity In Dependent Instrument (HEIDI) test to evaluate heterogeneity. Subsequently, mitochondrial-related genes were extracted at the levels of gene methylation, gene expression, and protein abundance levels. Causal inference was further strengthened through colocalization analysis. Finally, by combining the SMR results of gene methylation, gene expression, and protein abundance levels, we identified mitochondrial-related candidate genes causally associated with cholelithiasis. Additionally, to ensure the reliability of the analysis, no overlap existed between the exposure and outcome populations. We used publicly available summary-level data, therefore, ethical approval was not required. Figure 1 illustrates the overall design framework of this study.

### 2.1. Collections of QTL data for gene methylation, gene expression, and protein abundance levels

The integration of multi-omics data enables us to reveal the underlying molecular networks driving mitochondrial dysfunction. Among these, quantitative trait loci analysis is useful for identifying associations between single nucleotide polymorphisms (SNPs) and gene methylation, gene expression, and protein abundance levels. The SNP-CpG association data in blood were derived from McRae et al’s mQTL study (n = 1980).^[12]^ The blood gene expression quantitative trait locus (eQTL) dataset was derived from the eQTLGen consortium (n = 31,684).^[13]^ In addition, the Fenland database conducted a protein quantitative trait locus (pQTL) study using plasma samples from healthy individuals of European descent (n = 10,708),^[14]^ from which we extracted summary statistics on the genetic associations of circulating protein levels (Table 1).

**Table 1:** Information of included studies and consortia.

Exposure/outcome	Consortium/first author	Participants	Pubmed ID/Web source
mQTL	McRae et al	1980 European individuals	30514905
eQTL	eQTLGen Consortium	31,684 individuals (majority of samples were of European ancestry)	https://www.eqtlgen.org/cis-eqtls.html
pQTL	Pietzner et al	10,708 European individuals	34648354
Cholelithiasis	The FinnGen study	49,834 European-ancestry cases and 437,418 European-ancestry controls	https://www.finngen.fi/fi
	The UK Biobank study	43,639 European-ancestry cases and 506,798 European-ancestry controls	34651315

eQTL = expression quantitative trait loci, mQTL = methylation quantitative trait loci, pQTL = protein quantitative trait loci.

### 2.2. Collections of mitochondrial-related genes

This study utilized MitoCarta3.0 to identify mitochondrial-related genes. This database offers the latest list of human mitochondrial-related genes (n = 1136).^[15]^ MitoCarta used Bayesian method to integrate 7 experimental and sequence features, in order to systematically identify all protein components residing in the mitochondria. Additionally, MitoCarta3.0 independently reviews the literature for each mitochondrial-related gene to ensure its accuracy and reliability. Due to the limited pQTL data, after screening mitochondrial-related genes, we extracted only mitochondrial-associated methylation genes and expression genes from the mQTL and eQTL datasets.

### 2.3. Cholelithiasis outcome datasets

Summary-level data on cholelithiasis were obtained from the FinnGen study (n = 487,252), comprising 49,834 cholelithiasis cases and 437,418 controls, all of European descent. To validate our findings, we used GWAS data from the UK Biobank (n = 550,437), consisting of 43,639 cholelithiasis cases and 506,798 controls (Table 1).^[11]^ Notably, these 2 datasets are independent, with no overlapping samples.

### 2.4. SMR analysis

This study employed the SMR method to assess the association of mitochondrial gene methylation, gene expression, and protein abundance, with cholelithiasis risk.^[16]^ Cis-QTLs refer to DNA variant loci located on the same DNA strand as the gene they regulate and are in close proximity, typically within 1 Mb upstream or downstream of the gene, usually referring to SNPs in this region. SMR is based on top-ranked cis-QTLs (top-cis-QTLs), and when exposure and outcome come from 2 large independent samples, this method offers higher statistical power compared to traditional MR analysis.^[10]^ In this study, genes were centered within a ± 1000 kb window, and top-ranked cis-QTLs with a *P*-value < 5.0 × 10^−8^ were selected for analysis. The FinnGen (R12) GWAS dataset was filtered to exclude SNPs with allele frequency differences >0.05 (the threshold set in this study) to ensure data quality, including LD reference samples, QTL data, and GWAS results. Due to the limited GWAS data from the UK Biobank, SNPs with allele frequency differences >1 between datasets were excluded to ensure data integrity. The HEIDI test was used to distinguish pleiotropy and linkage. SNPs with P-HEIDI < .05 may exhibit pleiotropy and were excluded. SMR and HEIDI tests were performed using the SMR v1.3.1 software tool, and *P*-values were adjusted using the Benjamini–Hochberg method to control the false discovery rate (FDR) at ɑ = 0.05. After FDR adjustment, SNPs with *P*-value < .05 and P-HEIDI > .05 were used for subsequent colocalization analysis.

### 2.5. Colocalization analysis

Colocalization analysis was conducted using the coloc R package to detect common causal variants between cholelithiasis and identified mitochondrial-related mQTLs and eQTLs.^[16]^ The analysis was performed using default prior probabilities of p1 = 1E−4, p2 = 1E−4, and p12 = 1E−5, where p1, p2, and p12 represent the probability of an SNP being associated with trait 1, trait 2, and both traits, respectively. The colocalization posterior probability (PP) is categorized into 5 hypotheses: PPH0: The SNP within the colocalization region is associated with neither trait. PPH1: The SNP within the colocalization region is associated with the first trait but not the second. PPH2: The SNP within the colocalization region is associated with the second trait but not the first. PPH3: The SNP within the colocalization region is associated with both traits but at different loci. PP.H4: The SNP within the colocalization region is associated with both traits at the same locus. The significance threshold for colocalization was defined as PP.H4 > .50, and genes showing colocalization with cholelithiasis were considered potential therapeutic targets for cholelithiasis. Specifically, PP.H4 > .7 was considered strong colocalization, while .5 < PP.H4 ≤ .7 was considered moderate colocalization.

### 2.6. Classification of the causal mitochondrial-related genes with multi-omics evidence

To systematically investigate the causal relationship between mitochondria-associated genes and cholelithiasis, we integrated SMR results with colocalization analysis results for gene methylation, gene expression, and protein abundance. Based on the strength of evidence, mitochondria-associated pathogenic genes were classified into 2 categories: Tier 1 genes: Genes that exhibit a causal association with cholelithiasis at least at 2 omics levels (P_FDR < .05, P-HEIDI > .05) and are supported by colocalization evidence (PP.H4 > .5). Tier 2 genes: Genes that are associated with cholelithiasis at least at 2 omics levels (P_FDR < .05, P-HEIDI > .05) but have colocalization evidence at only a single omics level (PP.H4 > .5) or lack colocalization support (PP.H4 ≤ .5). These analytical methods are consistent with the previously described SMR and colocalization analyses, ensuring the reliability of the study findings.

### 2.7. Phenome-wide association study analysis

To assess the horizontal pleiotropy and potential side effects of candidate drug targets, this study conducted a phenome-wide association study (PheWAS) analysis through the AstraZeneca PheWAS Portal (https://azphewas.com/).^[17]^ The original PheWAS dataset was derived from the UK Biobank, consisting of 450,000 exome-sequenced individuals and encompassing approximately 15.5K binary phenotypes and 1.5K continuous phenotypes. A multiple testing correction was applied using a threshold of −log_10_(*P*) = 8. Genes exceeding this threshold were deemed associated with specific traits, suggesting potential side effects of the drug target.

### 2.8. Functional enrichment analysis

To investigate the potential biological functions of the identified mitochondrial-associated genes, we conducted Gene Ontology (GO) and Kyoto Encyclopedia of Genes and Genomes (KEGG) pathways enrichment analyses using “clusterProfiler” and “Pathview” R package.^[18]^ GO enrichment analysis is commonly used to depict the associations between genes and functional categories, including biological process (BP), molecular function (MF), and cellular component (CC). KEGG enrichment analysis elucidates the relationships between genes and functional pathways.^[19]^

### 2.9. Protein–protein interaction (PPI) network

By analyzing the PPI network, we can better understand how proteins interact within the cell. In this study, the PPI network was constructed using the STRING database, with a minimum interaction score of 0.4, keeping all other parameters at default settings.^[20]^ The PPI results were further visualized using Cytoscape (V3.9.1).^[21]^ Additionally, GeneMANIA (https://genemania.org/) was employed for PPI analysis.^[22]^

### 2.10. Drug prediction

Using the Drug Signatures Database (DSigDB), this study evaluated protein–drug interactions to predict candidate drugs.^[23]^ Specifically, DSigDB is a large database containing 22,527 gene sets and 17,389 distinct compounds covering 19,531 genes, associating drugs and other chemical compounds with their target genes. We uploaded the identified target genes to DSigDB, used this database to predict candidate drugs, and assessed their therapeutic potential.

### 2.11. Molecular docking

To gain deeper insights into the effects of candidate drugs on target genes and their therapeutic potential, this study performed molecular docking simulations at the atomic scale to evaluate the binding energy and interaction patterns between candidate drugs and their targets. Through molecular docking simulations, we examined the binding affinity between ligands and drug targets and explored their interaction patterns.

Ligands with high binding affinity and favorable interaction patterns were prioritized for further experimental validation and drug optimization. In this study, molecular structure data of candidate drugs were obtained from the PubChem Compound Database (https://pubchem.ncbi.nlm.nih.gov/), with ligand IDs listed in Table 5. Protein structure data were downloaded from the Protein Data Bank (PDB, http://www.rcsb.org/) and utilized as receptors for docking studies.^[24]^ Molecular docking and binding energy calculations were conducted using the CB-Dock2 web server. During preprocessing, all water molecules in the protein and ligand files were removed, and all nonrelevant isomers were discarded. The grid box was centered to encompass the protein domain, permitting unrestricted molecular movement. The study workflow is illustrated in Figure 1.

**Table 5:** Candidate drug predicted using DSigDB.

Drug names	*P*-value	Adjusted *P*-value	Genes
Indoxyl sulfate	.0040	.0572	HEBP1
Olmesartan	.0052	.0572	HEBP1
Lithocholic acid	.0052	.0572	HEBP1
Thioacetamide	.0054	.0572	HEBP1
Oleanolic acid	.0058	.0572	HEBP1
Fumaric acid	.0060	.0572	HEBP1
Buthionine sulfoximine	.0060	.0572	HEBP1
Neostigmine bromide PC3	.0061	.0572	TARS2; LIAS
Menadione sodium bisulfite	.0062	.0572	HEBP1
Bisacodyl PC3	.0064	.0572	TARS2

DSigDB = Drug Signatures Database.

## 3. Results

### 3.1. The causal relationship between mitochondrial-related genes methylation and cholelithiasis

**Figure 2.**
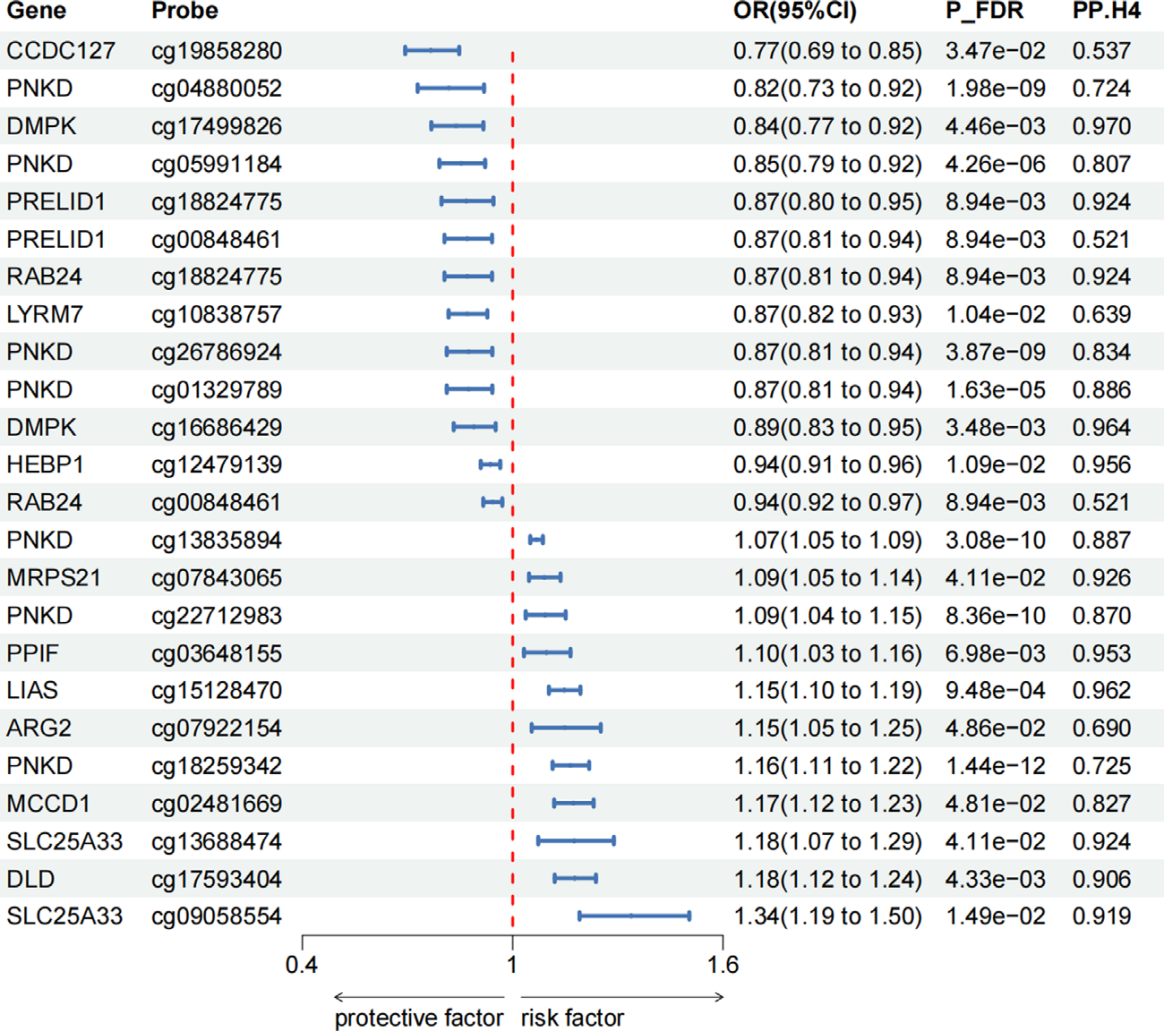
Associations of genetically predicted mitochondrial-related gene methylation with cholelithiasis in SMR analysis. (P_FDR < 0.05, P_HEIDI > 0.05, PP.H4 > 0.5). CI = confidence interval, FDR = false discovery rate, HEIDI = Heterogeneity In Dependent Instrument, OR = odds ratio, P_FDR = FDR-adjusted *P*-value, PP.H4 = posterior probability of H4, SMR = summary-based Mendelian randomization.

Using an extensive mQTL dataset derived from blood samples, we performed SMR analysis to explore the causal relationship between mitochondrial-related gene methylation and cholelithiasis. The SMR analysis encompassed 2430 CpG sites located near 664 genes. After applying multiple hypothesis testing (P_FDR < .05) and excluding horizontal pleiotropy (P_HEIDI > .05), we identified 57 CpG sites near 25 genes that are causally associated with cholelithiasis (Table S1, Supplemental Digital Content, https://links.lww.com/MD/P940). Of these, 24 CpG sites near 14 genes were supported by colocalization evidence (PP.H4 > .5) (Fig. 2).

Specifically, 19 CpG sites showed strong colocalization with cholelithiasis, including *DMPK* (cg17499826, cg16686429), *LIAS* (cg15128470), *HEBP1* (cg12479139), *PPIF* (cg03648155), *MRPS21* (cg07843065), *SLC25A33* (cg13688474, cg09058554), *PRELID1* (cg18824775), *RAB24* (cg18824775), *DL*D (cg17593404), *PNKD* (cg13835894, cg01329789, cg22712983, cg26786924, cg05991184, cg18259342, cg04880052), and *MCCD1* (cg02481669). Additionally, 5 CpG sites showed moderate colocalization, including *ARG2* (cg07922154), *LYRM7* (cg10838757), *CCDC127* (cg19858280), *PRELID1* (cg00848461), and *RAB24* (cg00848461).

Effect direction estimates were not always consistent for different CpG sites in the same gene. For example, in the *PNKD* gene, methylation of cg13835894 was associated with an increased risk of cholelithiasis (OR = 1.07, 95% CI = 1.05–1.09), while methylation of cg05991184 was associated with a decreased risk of cholelithiasis (OR = 0.85, 95% CI = 0.79–0.92) (Fig. 2).

Among the CpG sites after FDR adjustment and excluding horizontal pleiotropy, the associations of CpG sites near *PNKD* (cg13835894, cg22712983, cg01329789, cg26786924) were replicated in the UK Biobank. Additionally, the associations of cg17499826 and cg16686429 near *DMPK* were also replicated, and the association of cg02131230 near *MSRA* was validated (Table S2, Supplemental Digital Content, https://links.lww.com/MD/P940).

### 3.2. Causal relationship between mitochondrial-related genes expression and cholelithiasis

**Figure 3.**
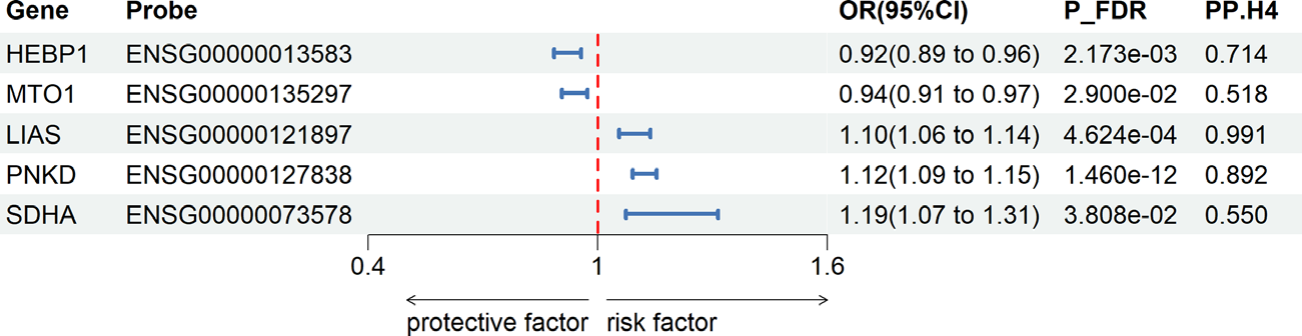
Associations of genetically predicted mitochondrial-related gene expression with cholelithiasis in SMR analysis. (P_FDR < 0.05, P_HEIDI > 0.05, PP.H4 > 0.5). CI = confidence interval, FDR = false discovery rate, HEIDI = Heterogeneity In Dependent Instrument, OR = odds ratio, P_FDR = FDR-adjusted *P*-value, PP.H4 = posterior probability of H4, SMR = summary-based Mendelian randomization.

To explore the causal relationship between mitochondrial-related gene expression and cholelithiasis, this study used eQTL data from blood samples of 31,684 participants for SMR analysis. This analysis included 1136 mitochondrial-related genes. Following FDR adjustment (P_FDR < .05) and excluding horizontal pleiotropy (P_HEIDI > .05), we identified 10 pathogenic genes (Table S3, Supplemental Digital Content, https://links.lww.com/MD/P940). Of these, 5 genes (*LIAS, HEBP1, PNKD, MTO1*, and *SDHA*) showed colocalization evidence (PP.H4 > .5), with *LIAS, HEBP1,* and *PNKD* showing stronger support, while *MTO1* and *SDHA* exhibited moderate support (Fig. 3).

Specifically, the expression of the following genes was associated with a decreased risk of cholelithiasis: *HEBP1* (OR = 0.92, 95% CI = 0.89–0.96) and *MTO1* (OR = 0.94, 95% CI = 0.91–0.97), suggesting that these genes may be related to a reduced risk of cholelithiasis. Conversely, the expression of the following genes was positively associated with cholelithiasis risk: *LIAS* (OR = 1.10, 95% CI = 1.06–1.14), *PNKD* (OR = 1.12, 95% CI = 1.09–1.15), and *SDHA* (OR = 1.19, 95% CI = 1.07–1.31). These genes’ expression may increase the risk of cholelithiasis.

Following multiple hypothesis testing (P_FDR < .05) and excluding horizontal pleiotropy (P_HEIDI > .05), the associations of ENSG00000121897 near *LIAS* and ENSG00000127838 near *PNKD* were replicated in the UK Biobank (Table S4, Supplemental Digital Content, https://links.lww.com/MD/P940). These results provide strong evidence for the potential role of mitochondrial-related genes in cholelithiasis and lay a foundation for further research and drug target development.

### 3.3. Causal relationship between mitochondrial-related plasma protein and cholelithiasis

In the discovery cohort, we utilized large-scale plasma pQTL data from Fenland Genetics and applied SMR and colocalization analysis to investigate the causal relationship between mitochondrial-related plasma proteins and cholelithiasis. For protein levels, only 5 plasma proteins showed borderline significance (*P* < .05) in association with cholelithiasis, with no evidence of horizontal pleiotropy (P_HEIDI > .05). However, after FDR adjustment, no significant associations were observed between these proteins and cholelithiasis risk (Table S5, Supplemental Digital Content, https://links.lww.com/MD/P940). This result may be explained by 2 factors: the development of pQTL data is still incomplete; there is a limited amount of cis-pQTL data that meet the requirements for SMR analysis.

**Figure 4.**
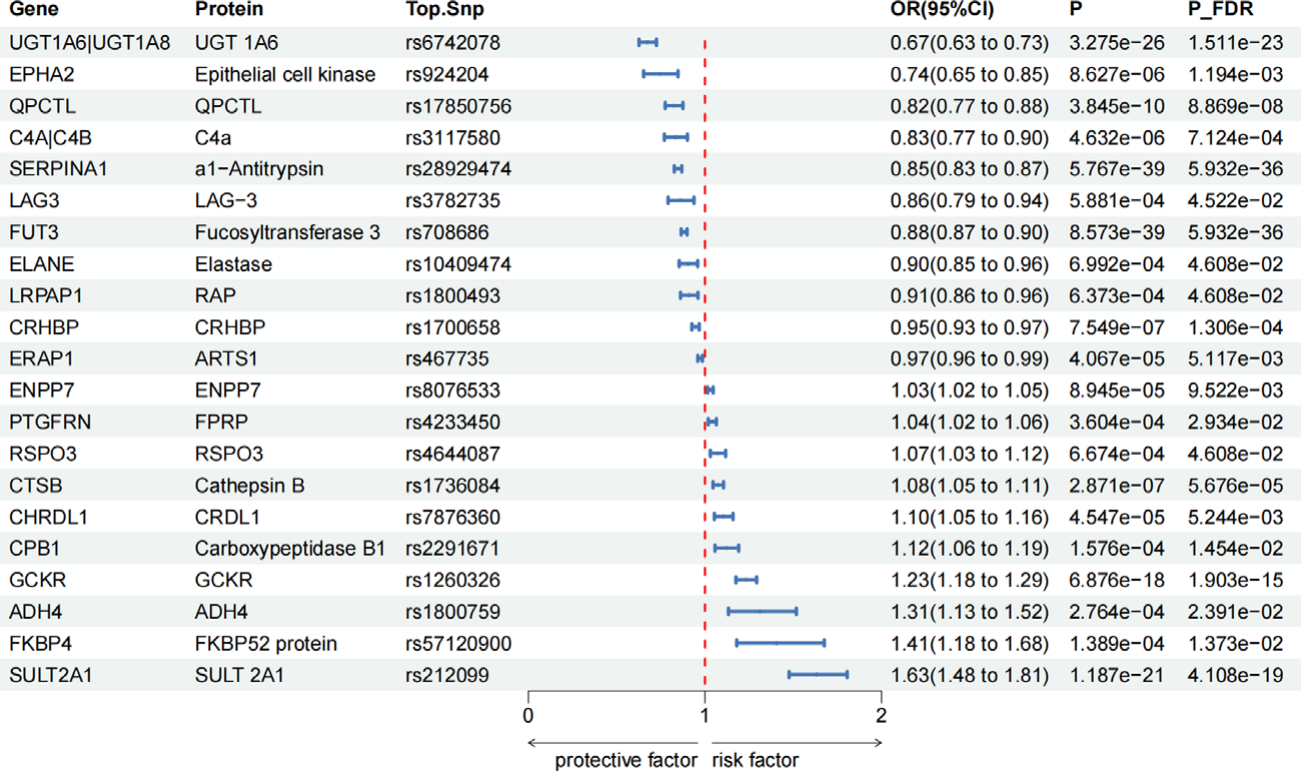
Associations of genetically predicted plasma protein levels with cholelithiasis in SMR analysis. (P_FDR < 0.05, P_HEIDI > 0.05, PP.H4 > 0.5). FDR = false discovery rate, HEIDI = Heterogeneity In Dependent Instrument, P_FDR = FDR-adjusted *P*-value, PP.H4 = posterior probability of H4, SMR = summary-based Mendelian randomization.

Additionally, for plasma proteins related to cholelithiasis, 173 proteins showed potential causal effects on cholelithiasis (*P* < .05) (Table S6, Supplemental Digital Content, https://links.lww.com/MD/P940). Among these, 21 plasma proteins maintained significant associations even after FDR adjustment (P_FDR < .05) (Fig. 4). For each standard deviation increase in genetically predicted plasma protein levels, the OR for causal effects ranged from 0.67 (95% CI = 0.63–0.73) for *UGT 1A6 | UGT 1A8* to 1.63 (95% CI = 1.48–1.81) for *SULT2A1* (Fig. 4).

Additionally, in the UK Biobank samples, we observed that 2 plasma proteins showed a significant association with cholelithiasis risk after FDR adjustment: *CROT* and *SPHK2* (Table S7, Supplemental Digital Content, https://links.lww.com/MD/P940). Specifically, genetically predicted higher levels of *CROT* (OR = 1.08, 95% CI = 1.04–1.12) were positively associated with an increased risk of cholelithiasis, while higher levels of *SPHK2* (OR = 0.75, 95% CI = 0.65–0.86) were associated with a decreased risk of cholelithiasis.

### 3.4. Integration of multi-omics analysis

In this study, since no significant protein–cholelithiasis associations remained after FDR correction in the discovery cohort (Table S5, Supplemental Digital Content, https://links.lww.com/MD/P940), we integrated the SMR and colocalization analysis results of gene methylation and gene expression with cholelithiasis. As a result, we identified 4 mitochondrial-related genes associated with cholelithiasis risk (Table 2).

**Table 2:** Associations of genetically predicted gene methylation, gene expression of candidate genes with cholelithiasis.

Gene	Tier	mQTL-cholelithiasis				eQTL-cholelithiasis			
		Probe	OR (95%Cl)	P_FDR	PP.H4	Probe	OR (95%Cl)	P_FDR	PP.H4
LIAS	Tier 1	cg15128470	0.936 (0.909–0.964)	.00094827	9.62E−01	ENSG00000121897	1.096 (1.056–1.138)	4.62E−04	9.91E−01
HEBP1	Tier1	cg12479139	0.839 (0.768–0.916)	.01092504	9.56E−01	ENSG00000013583	0.920 (0.886–0.956)	2.17E−03	7.14E−01
PNKD	Tier1	cg05991184	0.766 (0.693–0.847)	.00000426	8.07E−01	ENSG00000127838	1.122 (1.091–1.154)	4.26E−06	8.07E−01
		cg04880052	1.173 (1.118–1.230)	1.98E−09	7.24E−01				
		cg26786924	1.177 (1.120–1.237)	3.87E−09	8.34E−01				
		cg01329789	1.338 (1.191–1.504)	.0000163	8.86E−01				
		cg18259342	1.068 (1.050–1.086)	1.44E−12	7.25E−01				
		cg22712983	1.165 (1.114–1.218)	8.36E−10	8.70E−01				
		cg13835894	1.147 (1.103–1.193)	3.08E−10	8.87E−01				
TARS2	Tier2	cg09579323	1.053 (1.021–1.085)	.041061437	1.04E−01	ENSG00000143374	0.728 (0.616–0.860)	1.38E−02	2.28E−03

eQTL = expression quantitative trait loci, FDR = false discovery rate, mQTL = methylation quantitative trait loci, P_FDR = FDR-adjusted *P*-value, PP.H4 = posterior probability of H4.

In this study, *LIAS, HEBP1*, and *PNKD* were designated as Tier 1 genes. Specifically, the methylation of cg15128470 in *LIAS* (OR = 0.936, 95% CI = 0.909–0.964) was associated with a reduced risk of cholelithiasis. Similarly, methylation at cg12479139 in *HEBP1* (OR = 0.839, 95% CI = 0.768–0.916) and at cg05991184 in *PNKD* (OR = 0.766, 95% CI = 0.693–0.847) were also protective factors against cholelithiasis (Table 3). It is noteworthy that methylation at 6 additional CpG sites in *PNKD* was linked to an increased risk of cholelithiasis.

**Table 3:** Correlativity of genetically predicted gene methylation, gene expression of candidate genes with cholelithiasis.

Gene	Tier	mQTL-cholelithiasis		eQTL-cholelithiasis	
		Probe	Correlativity	Probe	Correlativity
LIAS	Tier1	cg15128470	↓	ENSG00000121897	↑
HEBP1	Tier1	cg12479139	↓	ENSG00000013583	↓
PNKD	Tier1	cg05991184	↓	ENSG00000127838	↑
		cg04880052	↑		
		cg26786924	↑		
		cg01329789	↑		
		cg18259342	↑		
		cg22712983	↑		
		cg13835894	↑		
TARS2	Tier2	cg09579323	↑	ENSG00000143374	↓

↓: negative correlation, ↑: positive correlation.

eQTL = expression quantitative trait loci, mQTL = methylation quantitative trait loci.

Additionally, increased gene expression of *LIAS* (OR = 1.10, 95% CI = 1.06–1.14) and *PNKD* (OR = 1.12, 95% CI = 1.09–1.15) in blood was associated with an increased risk of cholelithiasis, while higher expression of *HEBP1* (OR = 0.92, 95% CI = 0.89–0.96) was associated with a reduced risk of cholelithiasis. The results of colocalization analysis provided strong evidence for the causal relationship between Tier 1 genes and cholelithiasis.

*TARS2* was designated as a Tier 2 gene. Methylation of cg09579323 in *TARS2* (OR = 1.05, 95% CI = 1.02–1.08) was associated with an increased risk of cholelithiasis, whereas increased *TARS2* gene expression (OR = 0.73, 95% CI = 0.62–0.86) was associated with a reduced cholelithiasis risk.

To further investigate the relationship between gene methylation and gene expression, we performed SMR and colocalization analyses (Table 4). The results showed that methylation of cg15128470 in *LIAS* was linked to reduced *LIAS* expression, aligning with its protective role against cholelithiasis. Conversely, methylation of cg12479139 in *HEBP1* was positively correlated with gene expression, consistent with previous findings.

**Table 4:** Association between methylation and expression of mitochondrial-related genes.

Gene	Tier	mQTL–eQTL					
		Probe	Beta	SE	P_FDR	P-HEIDI	PP.H4
LIAS	Tier1	cg15128470	−0.431156	0.02947507	1.14E−46	.999353825	9.94E−01
HEBP1	Tier1	cg12479139	1.401883911	0.22507054	4.31E−09	.966194274	.317
PNKD	Tier1	cg05991184	−2.17118096	0.315354628	1.24E−10	.22091406	7.23E−01
		cg04880052	1.297761367	0.11286441	8.48E−29	.057342282	9.88E−01
		cg26786924	1.435241437	0.130870763	5.69E−26	.573116809	9.91E−01
		cg01329789	2.289501509	0.373090452	9.88E−09	.746647331	9.45E−01
		cg18259342	0.535780792	0.017955928	6.43E−193	.999999882	1.00E + 00
		cg22712983	1.322291088	0.109561905	5.20E−31	.941844945	9.99E−01
		cg13835894	1.0371205	0.07916174	4.55E−38	.999935764	9.59E−01
TARS2	Tier2	cg09579323	−0.15342348	0.01998904	1.01E−12	.993221476	9.71E−01

eQTL = expression quantitative trait loci, FDR = false discovery rate, HEIDI = Heterogeneity In Dependent Instrument, mQTL = methylation quantitative trait loci, P_FDR = FDR-adjusted *P*-value, PP.H4 = posterior probability of H4.

In *PNKD*, except for cg05991184, whose methylation was negatively correlated with RNA expression, the methylation of the remaining 6 CpG sites was positively correlated with RNA expression.

Among the Tier 2 genes, methylation of cg09579323 in *TARS2* was associated with decreased *TARS2* expression. These findings support their proposed causal roles in cholelithiasis.

Except for *HEBP1* (PP.H4 = 3.17E−01), all other genes demonstrated strong support in the mQTL-eQTL relationship (PP.H4 > .7), further strengthening the reliability of their causal associations.

### 3.5. PheWAS analysis of the causal mitochondrial-related genes

To further assess whether the 4 identified potential drug target genes exert favorable or adverse effects on other traits and to identify any potential pleiotropy not detected by P_HEIDI and colocalization analysis, this study used data from the AstraZeneca PheWAS Portal. This database consists of 17,361 binary phenotypes and 1419 quantitative phenotypes.^[17]^ This study performed a gene-level PheWAS to explore the association between genetically determined protein expression and specific diseases or traits.

**Figure 5.**
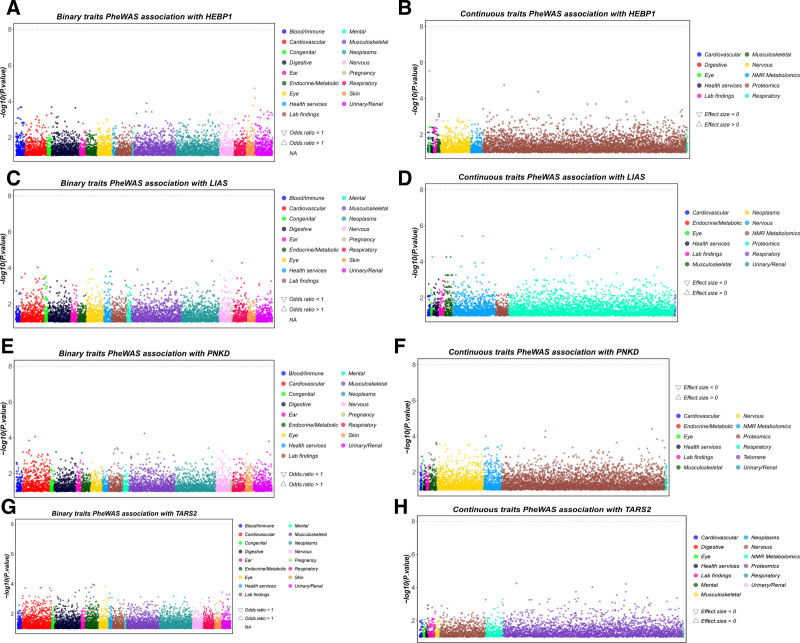
The association between genetically determined protein expression and specific traits. (A) The association between *HEBP1* gene-determined protein expression and the binary trait. (B) The association between *HEBP1* gene-determined protein expression and the continuous trait. (C) The association between *LIAS* gene-determined protein expression and the binary trait. (D) The association between *LIAS* gene-determined protein expression and the continuous trait. (E) The association between *PNKD* gene-determined protein expression and the binary trait. (F) The association between *PNKD* gene-determined protein expression and the continuous trait. (G) The association between *TARS2* gene-determined protein expression and the binary trait. (H) The association between *TARS2* gene-determined protein expression and the continuous trait. [Binary traits encompassed 17 clinically defined categories: blood/immune, mental, cardiovascular, musculoskeletal congenital, neoplasms, digestive, nervous, ear, pregnancy, endocrine/metabolic, respiratory, eye, skin, health services, urinary/renal and lab findings. Continuous traits encompassed 13 quantitative domains: cardiovascular, neoplasms, digestive, nervous, eye, NMR metabolomics, health services, proteomics, lab findings, respiratory, mental, urinary/renal and musculoskeletal.]

As illustrated in Figure 5 (comprising 8 subfigures, Fig. 5A–H), Figure 5A and B presents the association between *HEBP1* gene-determined protein expression levels and binary/continuous traits, respectively. Similarly, Figure 5C to F demonstrates corresponding relationships for *LIAS* and *PNKD* genes, while Figure 5G and H depicts *TARS2* gene–protein associations. Statistical analysis revealed no significant associations between these 4 drug target genes and any measured traits (both binary and continuous) at the predefined significance threshold [−log10(*P*.value) < 8]. This finding suggested a reduced risk of side effects for these drug targets and a lower likelihood of horizontal pleiotropy, further reinforcing the robustness of our findings.

### 3.6. Enrichment analysis: KEGG and GO enrichment analysis

**Figure 6.**
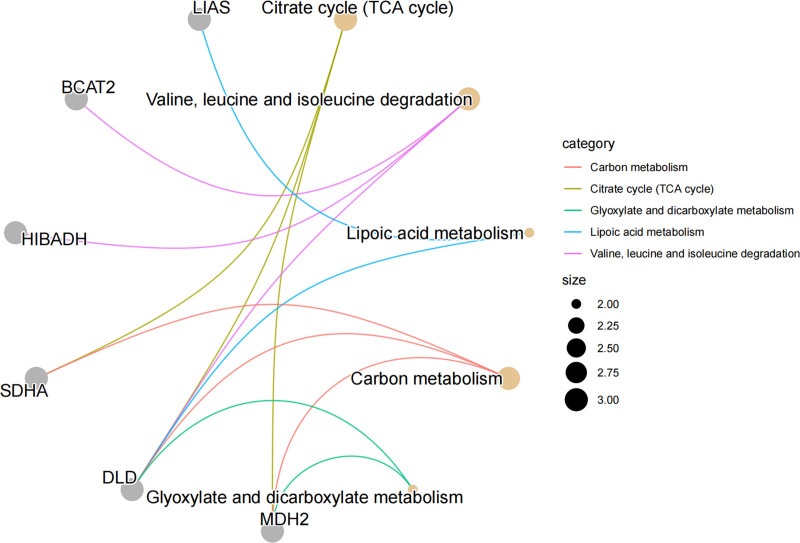
Pathway enrichment analysis of the identified mitochondrial-related genes: the plot showed the enrichment results of KEGG pathways. KEGG = Kyoto Encyclopedia of Genes and Genomes.

GO enrichment analysis is commonly used to depict the associations between genes and BPs or functional categories, while KEGG enrichment analysis elucidates the roles of genes within functional pathways.^[19]^ The KEGG enrichment analysis in this study revealed the top 3 enriched pathways as the tricarboxylic acid (TCA) cycle, valine, leucine, and isoleucine degradation, and carbon metabolism (Fig. 6).

The tricarboxylic acid (TCA) cycle, also known as the citric acid cycle or Krebs cycle, is a fundamental metabolic pathway universally present in aerobic organisms and serves as the central hub of cellular metabolism. It plays a critical role in nutrient oxidation and ATP generation, supplying energy for cellular processes.^[25]^ Additionally, the TCA cycle not only serves as the terminal metabolic pathway for carbohydrates, lipids, and amino acids but also acts as a central hub for the interconversion of various organic compounds. Certain intermediates of the cycle participate in physiological processes, including immune regulation.^[26]^ This study hypothesizes that the TCA cycle may be involved in the onset and progression of cholelithiasis by regulating lipid metabolism, energy metabolism, and oxidative stress pathways.

Notably, valine, leucine, and isoleucine are essential branched-chain amino acids, and their metabolic dysregulation is closely associated with insulin resistance. Insulin resistance is considered a potential risk factor for cholelithiasis.^[27]^

Carbon metabolism involves the metabolic processes of carbohydrates in the human body. Previous studies have shown a direct relationship between high-carbohydrate intake and gallstone formation. A high-carbohydrate diet, often accompanied by reduced fat intake, may lead to decreased cholecystokinin secretion, resulting in reduced gallbladder motility. This, in turn, can cause bile stasis and increase the risk of gallstone development.^[28]^

**Figure 7.**
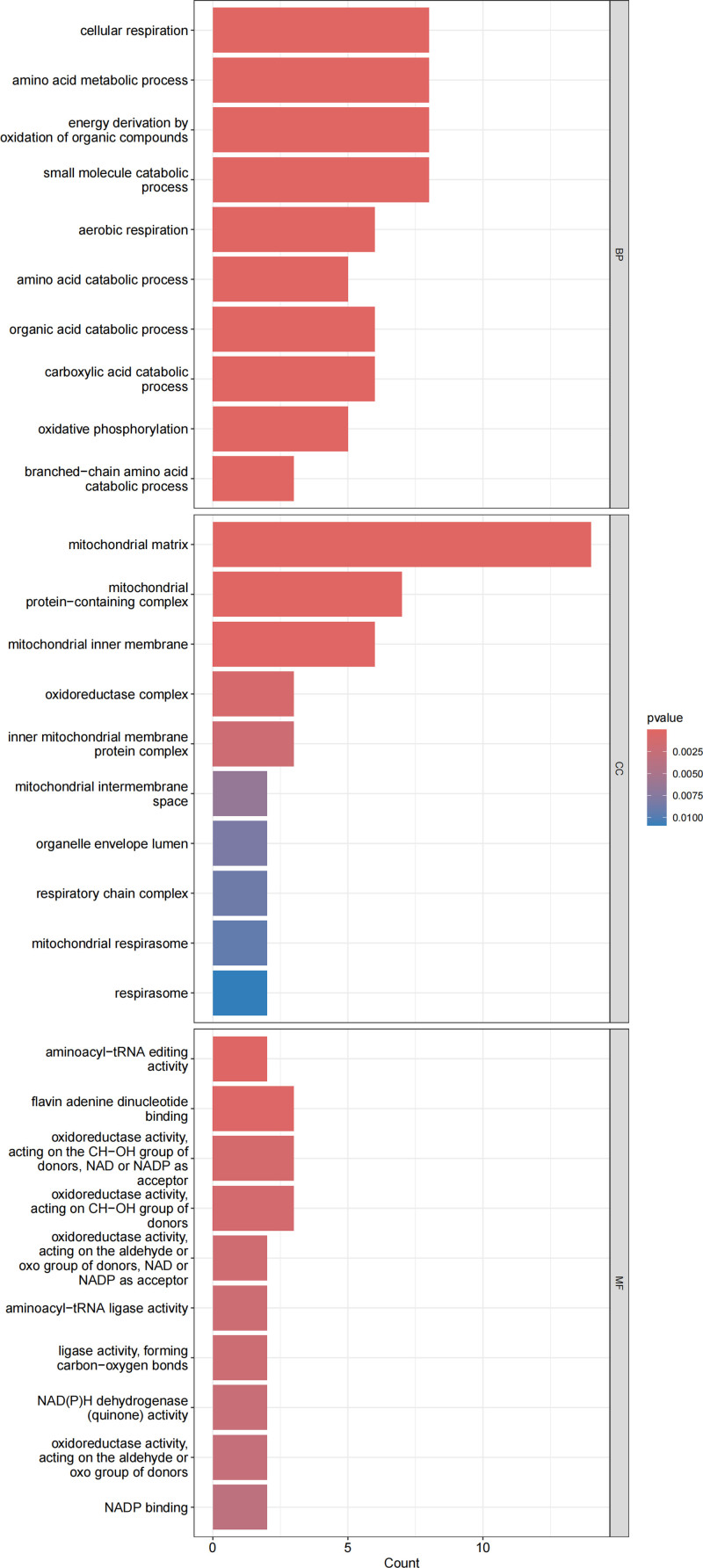
Pathway enrichment analysis of the identified mitochondrial-related genes: the plot displayed the enrichment results of GO pathways. GO = gene ontology.

GO enrichment analysis consists of 3 categories: BP, CC, and MF. As shown in Figure 7, within the BP category, these genes are primarily involved in nutrient metabolism and energy production processes, including cellular respiration, amino acid metabolism, organic compound oxidation for energy generation, and small molecule catabolism. In the CC category, these drug target genes are significantly enriched in mitochondrial-related components, including mitochondrial structure, mitochondrial complexes, and the inner mitochondrial membrane. These findings suggest that cholelithiasis-associated genes may primarily function within mitochondria, influencing cellular metabolism and energy conversion. Within the MF category, the most significantly enriched pathways are predominantly associated with redox processes, mainly including oxidoreductase activity (acting on the CH–OH group as a donor, with NAD or NADP as an acceptor; acting on the CH–OH group).

Overall, the results of GO enrichment analysis are consistent with those of KEGG enrichment analysis, both indicating that cholelithiasis-associated genes play a crucial role in substance metabolism and energy metabolism. These findings further support the hypothesis that mitochondrial dysfunction may contribute to the pathogenesis and progression of cholelithiasis.

### 3.7. PPI network

**Figure 8.**
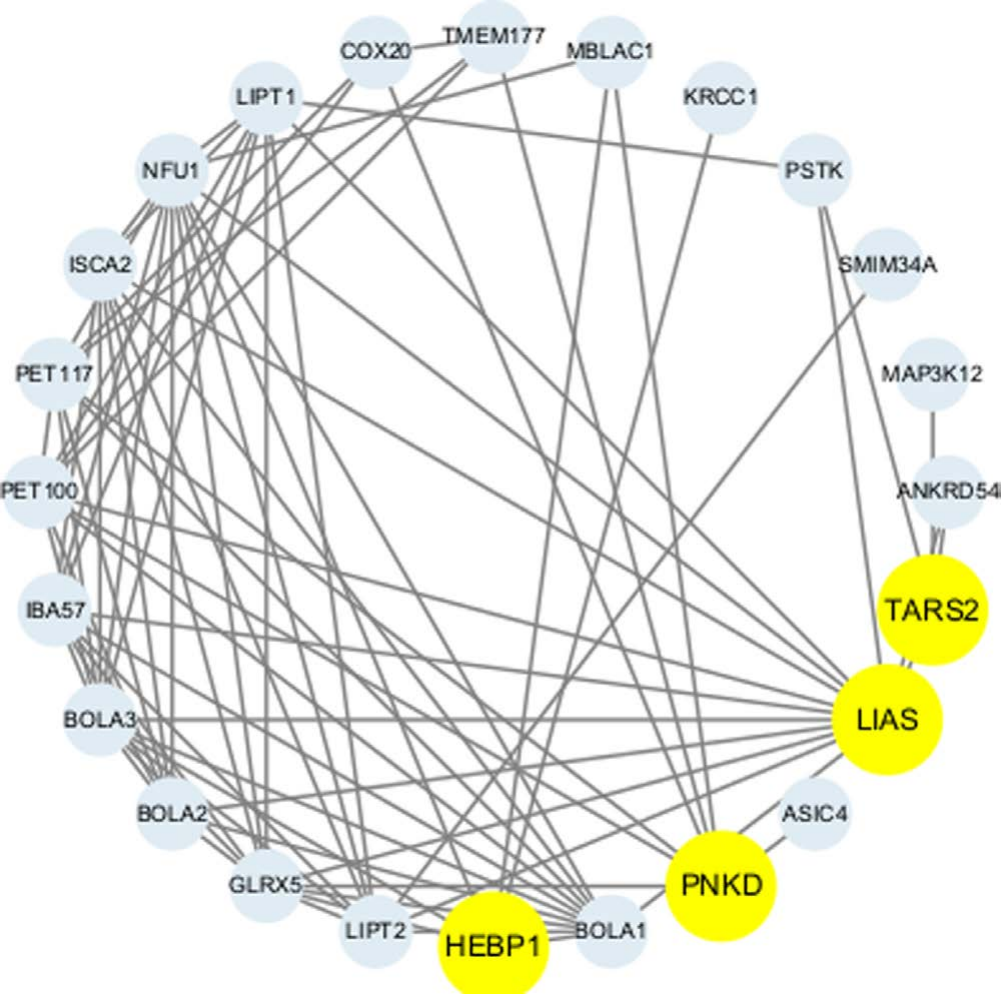
PPI network built with STRING. PPI = protein–protein interaction networks.

We uploaded the 4 drug target genes into the STRING database (https://cn.string-db.org/) to construct an interaction network and imported the generated files into Cytoscape for visualization. Figure 8 presents a PPI network containing 24 nodes and 75 edges, illustrating the interactions between the 4 drug target genes and other proteins.

**Figure 9.**
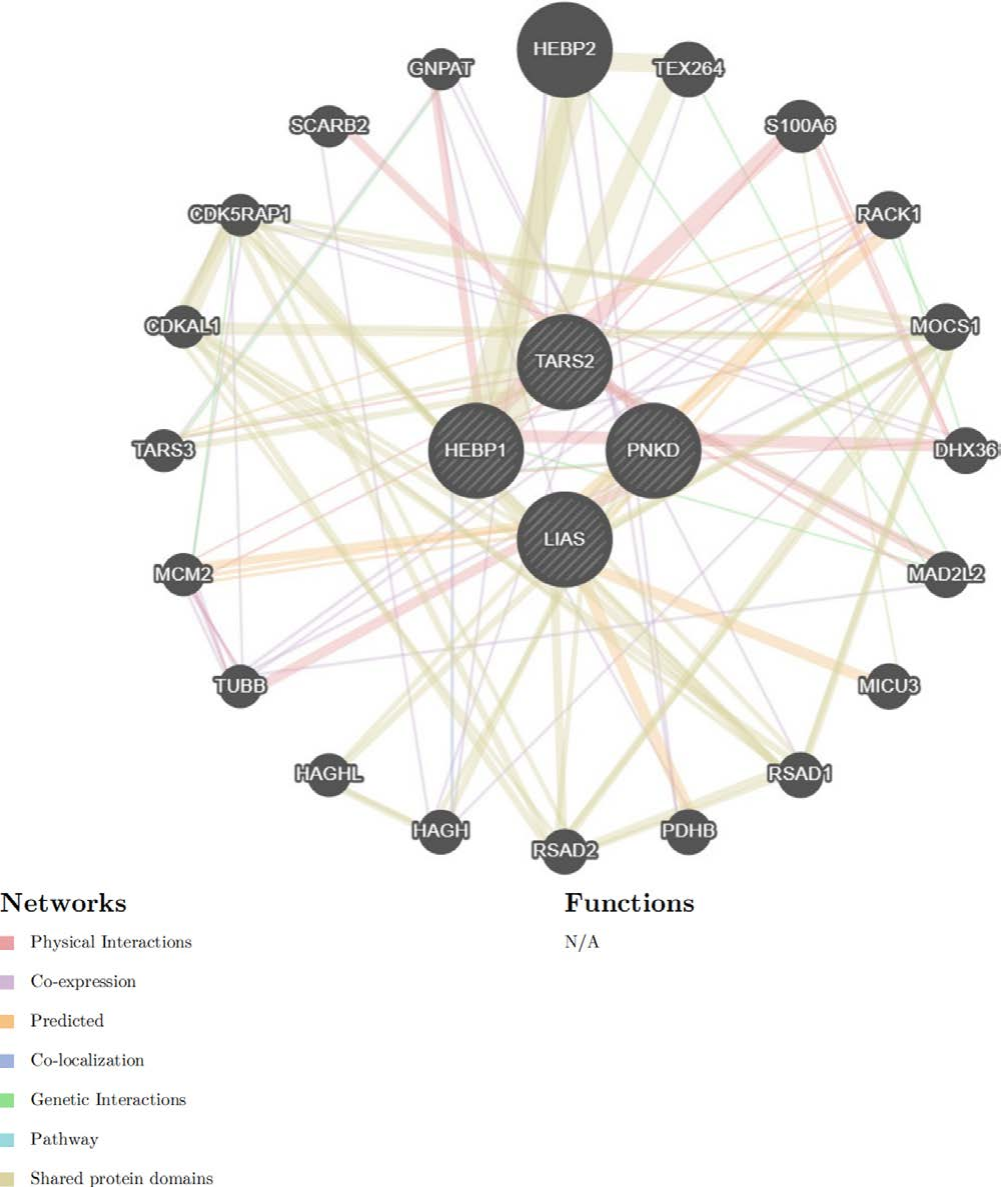
PPI network built with GeneMANIA (each circle is colored to indicate the functional pathway in which each gene is involved). PPI = protein–protein interaction networks.

Additionally, a PPI network was constructed using GeneMANIA (https://genemania.org/), which included not only the 4 drug target genes but also 20 additional potentially interacting genes, generating a total of 106 interaction links (Fig. 9). The network visualizations depict the direct relationships among these genes, encompassing physical interactions, co-expression, predicted interactions, colocalization, genetic interactions, pathway associations, and shared protein domains.

### 3.8. Candidate drug prediction

This study utilized DSigDB from the Enrichr database to predict potential therapeutic compounds. Based on the adjusted *P*-value, the top 10 candidate compounds were identified (Table 5), where a smaller *P*-value indicates a stronger association between the drug and its corresponding target gene.

The results showed that bisacodyl PC3 and neostigmine bromide PC3(“PC3” is a specific formulation of the drug) were the 2 most significant drugs associated with *TARS2*. Additionally, neostigmine bromide was also identified as the most significant drug for *LIAS.* In contrast, *HEBP1* exhibited interactions with a broader range of drugs.

### 3.9. Molecular docking

**Figure 10.**
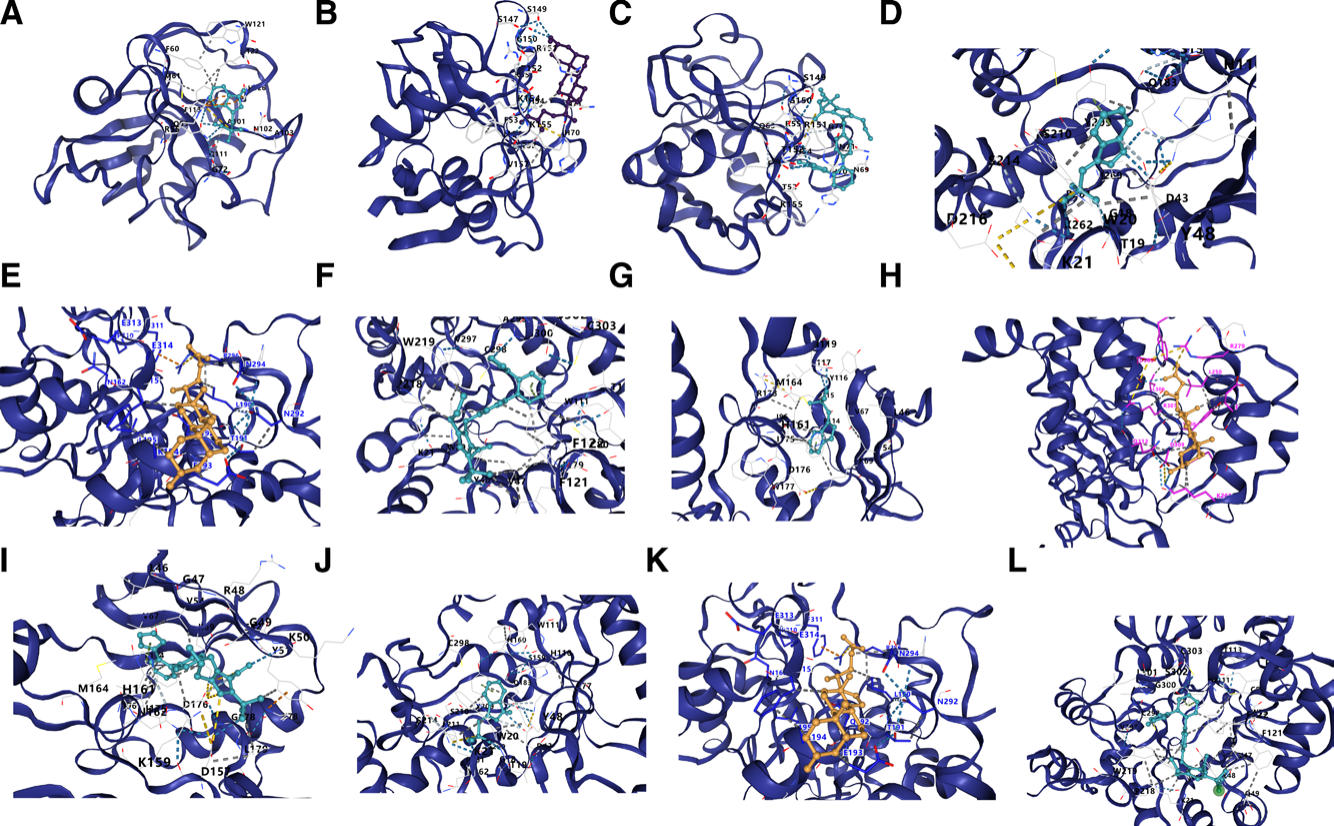
Docking results of available proteins small molecules. (A) *HEBP1* docking Indoxyl sulfate, (B) *HEBP1* docking lithocholic acid, (C) *HEBP1* docking Olmesartan, (D) *LIAS* docking Indoxyl sulfate, (E) *LIAS* docked to lithocholic acid, (F) *LIAS* docked to Olmesartan, (G) *PNKD* docking Indoxyl sulfate, (H) *PNKD* docking lithocholic acid, (I) *PNKD* docking Olmesartan, (J) *TARS2* docking Indoxyl sulfate, (K) *TARS2* docking lithocholic acid, (L) *TARS2* docking Olmesartan.

To evaluate the binding affinity of candidate drugs to their targets and further assess their druggability, this study conducted molecular docking analysis. Using the CB-Dock2 online platform, we identified the top 3 candidate drugs, mapped their binding sites with the corresponding gene-encoded proteins, and calculated the binding energy for each interaction (Table 6). A total of 12 effective protein–drug docking results were obtained (Fig. 10A–10L).

**Table 6:** The binding energies of the first 3 candidate drugs interacting with the corresponding gene-encoded proteins.

Drug	Protein	Binding energy
Indoxyl sulfate	HEBP1	−5.5
Olmesartan	HEBP1	−6.7
Lithocholic acid	HEBP1	−6.5
Indoxyl sulfate	TARS2	−8
Olmesartan	TARS2	−9
Lithocholic acid	TARS2	−9.2
Indoxyl sulfate	LIAS	−8
Olmesartan	LIAS	−9.2
Lithocholic acid	LIAS	−6.2
Indoxyl sulfate	PNKD	−7.7
Olmesartan	PNKD	−10.1
Lithocholic acid	PNKD	−8.4

Each candidate drug interacted with its protein target through hydrogen bonds and strong electrostatic interactions. Furthermore, all 3 candidate drugs successfully occupied the binding pockets of their respective targets. Among them, *PNKD* and olmesartan exhibited the lowest binding energy (−10.1 kcal/mol), indicating a highly stable interaction.

## 4. Discussion

This study, through multi-omics integrative analysis, provides the first evidence of a causal link between mitochondrial-related genes (*LIAS, TARS2, HEBP1, PNKD*) and cholelithiasis, and evaluates their potential as viable drug targets. The following points discuss the biological significance and underlying mechanisms of these key genes, in light of our findings and existing literature:

### 4.1. *LIAS* gene: imbalance in lipoic acid synthesis and energy metabolism

*LIAS* encodes the rate-limiting enzyme for the synthesis of α-lipoic acid (1,2-dithiolane-3-pentanoic acid), which is produced in the mitochondria. It is a natural antioxidant and an essential cofactor in mitochondrial α-ketoacid dehydrogenase complexes, such as the pyruvate dehydrogenase complex and α-ketoglutarate dehydrogenase complex. Both of these enzyme complexes are involved in glucose oxidation and the synthesis of adenosine triphosphate (ATP).^[29]^ This study found that elevated methylation of *LIAS* (cg15128470) reduces the risk of cholelithiasis by inhibiting gene expression. Molecular docking also revealed that olmesartan interacts strongly with *LIAS* (−9.2 kcal/mol), suggesting that it may modulate cholelithiasis risk by regulating the lipoic acid metabolic pathway, although the exact mechanism requires further experimental validation.

### 4.2. *TARS2* gene: mitochondrial protein synthesis and indirect metabolic effects

*TARS2* encodes asparaginyl-tRNA synthetase, which plays a crucial role in protein synthesis. Although no colocalization evidence for *TARS2* and cholelithiasis was found in this study (Tier 2 gene), reduced expression of *TARS2* was associated with a lower risk of cholelithiasis (OR = 0.73, 95% CI = 0.62–0.86). Methylation of cg09579323 downregulates *TARS2* expression, thereby increasing cholelithiasis risk, suggesting that *TARS2* may indirectly regulate bile acid synthesis enzymes by maintaining mitochondrial translation efficiency.

Moreover, molecular docking revealed that lithocholic acid (LCA) binds tightly to *TARS2* (binding energy: −9.2 kcal/mol). The liver converts cholesterol into primary bile acids, which enter the intestine and undergo further transformation by gut microbiota. Cholic acid (CA) can be converted into deoxycholic acid (DCA), while chenodeoxycholic acid (CDCA) can be metabolized into LCA, which is further processed by microbial enzymes in the gut.^[30]^ This suggests that enhancing the metabolism of LCA may indirectly facilitate gallstone dissolution.

Drug prediction identified bisacodyl as a potential therapeutic agent targeting *TARS2*. Bisacodyl is a contact laxative that acts on sensory nerve endings in the intestinal mucosa, thereby enhancing reflex peristalsis and promoting bowel movements. This suggests that bisacodyl may exert a similar effect on gallbladder smooth muscle, potentially promoting bile secretion and reducing gallstone formation.^[31]^ Neostigmine bromide has been identified as a candidate drug for cholelithiasis targeting the *LIAS* and *TARS2* genes. As an acetylcholinesterase inhibitor, it is commonly used to treat postoperative paralytic ileus and urinary retention caused by abdominal surgery. This suggests that acetylcholinesterase inhibitors may promote gallbladder emptying by activating cholinergic signaling; however, their potential pleiotropic side effects should be carefully considered.^[32]^

### 4.3. *HEBP1* gene: regulation of lipid metabolism and bile composition homeostasis

The *HEBP1* gene, fully named *heme binding protein 1* gene, encodes heme-binding protein 1, a mitochondria-localized lipid-binding protein involved in fatty acid transport and membrane stability maintenance.^[33]^ This study demonstrates that both *HEBP1* methylation and *HEBP1* gene expression significantly reduce the risk of cholelithiasis. This finding supports the role of *HEBP1* in maintaining mitochondrial membrane integrity and promoting the balance of phospholipids and cholesterol in bile, thereby inhibiting cholesterol crystallization. Notably, molecular docking revealed a relatively low binding energy between *HEBP1* and olmesartan (−6.7 kcal/mol), which may be associated with its nonclassical target interactions. Further functional studies are required to elucidate its pharmacological mechanism.

### 4.4. *PNKD* gene: biliary dynamics and gallbladder contractility

The *PNKD* gene, also known as *MR-1 (Myofibrillogenesis Regulator 1*), is associated with paroxysmal non-kinesigenic dyskinesia (PNKD). Its protein may influence smooth muscle contraction by modulating calcium ion channels.^[34]^ This study found that elevated *PNKD* expression (OR = 1.12) significantly increases the risk of cholelithiasis, whereas methylation at specific CpG sites (cg05991184, OR = 0.85) exhibits a protective effect. This paradox may reflect the bidirectional regulation of *PNKD*: increased gene expression may inhibit gallbladder smooth muscle contraction, leading to bile stasis, whereas specific methylation modifications might mitigate this effect through epigenetic silencing. Molecular docking revealed a high binding affinity between *PNKD* and olmesartan (−10.1 kcal/mol), suggesting that this drug may improve gallbladder motility by targeting *PNKD*. However, further experimental validation is needed to elucidate its precise regulatory network.

### 4.5. Pathway integration and translational significance

KEGG enrichment analysis revealed that the TCA cycle and branched-chain amino acid metabolism are the key regulatory pathways. Based on previous studies, mitochondrial dysfunction may contribute to cholelithiasis through the following mechanisms: Disruption of the TCA cycle leads to an imbalance in the NADH/NAD⁺ ratio, activating the sterol regulatory element-binding protein (SREBP) pathway and upregulating cholesterol biosynthesis.^[35]^ Impaired branched-chain amino acid degradation induces insulin resistance, which indirectly increases bile cholesterol saturation.^[27]^

The novelty of this study lies in the development, for the first time, of a causal pathway linking mitochondria-related genes, metabolic pathways, and cholelithiasis through multi-omics integration, providing theoretical support for personalized therapies targeting specific genes like *LIAS* and *HEBP1*. However, there are several limitations to this study. First, due to the limited number of mitochondrial-related proteins in the pQTL dataset, the current study has not fully explored the causal relationship between mitochondrial proteins and cholelithiasis risk. Second, the colocalization analysis should be interpreted with caution, particularly with respect to the PP.H4. When PPH3 is low due to insufficient statistical power, a low PP.H4 does not imply a lack of colocalization evidence.^[16]^ Third, the diversity of the study cohort is a limitation. Although the mQTL and eQTL analyses included individuals of non-European ancestry, the cholelithiasis cohort was composed solely of individuals of European descent. Due to differences in genetic backgrounds and patterns of linkage disequilibrium, this population bias may introduce potential bias in the SMR effect estimates. Therefore, extrapolation of the findings to individuals of other ethnicities requires further validation to ensure their broad applicability. In addition, the eQTL analysis primarily focused on cis-eQTLs and their association with cholelithiasis, potentially overlooking the impact of other regulatory elements and environmental factors on disease complexity. Although efforts were made to reduce bias, SMR analysis remains susceptible to unmeasured confounding factors or pleiotropy, and we must acknowledge these limitations and their potential impact on the conclusions. While enrichment analysis helps reveal biological significance, it also has limitations, as it relies on predefined gene sets or pathways, which may not comprehensively capture all biological mechanisms or interactions. Nonsignificant enrichments do not imply a lack of biological relevance, and researchers should interpret the results with caution. Finally, the accuracy of molecular docking analysis largely depends on the quality of protein structures and ligands. To our knowledge, there is currently no direct evidence supporting that olmesartan or neostigmine bromide alters the composition of bile acids. Olmesartan is primarily excreted via biliary transporters (e.g., MRP2) without affecting bile acid species or metabolism.^[36]^ Neostigmine bromide acts through cholinergic stimulation to facilitate gallbladder contraction and bile flow,^[37]^ but has not been linked to changes in bile acid composition. LCA, a secondary bile acid derived from chenodeoxycholic acid, has been identified and quantified in metabolomic studies of liver injury and bile acid disorders. In drug-induced liver injury patients, serum levels of LCA were significantly reduced in more severe cases.^[38]^ These findings highlight the need for further experimental validation to explore the possible effects of these drugs on bile acid metabolism and composition.

To further understand the pathogenesis of cholelithiasis and potential therapeutic approaches, we must acknowledge and address these limitations, laying the foundation for future research. Integrating omics data from diverse populations and exploring alternative analytical methods will help advance the field with a more comprehensive perspective.

## 5. Conclusion

This study, using the SMR and HEIDI testing methods, investigated the association of genetically predicted mitochondrial-related gene methylation, gene expression, and cholelithiasis, identifying 4 potential drug targets for cholelithiasis: *TARS2, LIAS, HEBP1,* and *PNKD*. This approach effectively controls for measurement errors and confounding factors, while colocalization analysis provides robust additional evidence. To explore the pleiotropy of these target genes and assess potential drug side effects, we also conducted PheWAS. Moreover, we performed enrichment analysis and PPI network analysis to further elucidate the biological significance of these targets. Lastly, we predicted drugs targeting these genes and conducted molecular docking to validate their druggability. Through multi-omics integration, we have constructed for the first time a causal pathway linking mitochondrial-related genes, metabolic pathways, and cholelithiasis, providing theoretical support for personalized therapies targeting the genes *LIAS, TARS2, HEBP1,* and *PNKD.*

## Acknowledgments

We thank all participants and investigators involved in the McRae et al genome-wide association analysis on methylation, the eQTLGen consortium, Pietzner et al genome-wide association analysis on the proteome, the FinnGen study, and the UK Biobank study for sharing data.

## Author contributions

**Conceptualization:** Liying Zhu.

**Data curation:** Haiyan Hou.

**Formal analysis:** Haiyan Hou.

**Methodology:** Haiyan Hou.

**Software:** Haiyan Hou.

**Supervision:** Zhuyi Jiang, Liying Zhu.

**Writing – original draft:** Haiyan Hou.

**Writing – review & editing:** Haiyan Hou.

## Supplementary Material



## References

[1] Constantinescu T Huwood Al Jabouri AK Brãtucu E Olteanu C Toma M Stoiculescu A. Gallstone disease in young population: incidence, complications, therapeutic approach. Chirurgia (Bucur). 2012;107:579–82. 23116830

[2] García-Berumen CI Ortiz-Avila O Vargas-Vargas MA. The severity of rat liver injury by fructose and high fat depends on the degree of respiratory dysfunction and oxidative stress induced in mitochondria. Lipids Health Dis. 2019;18:78. 30927921 10.1186/s12944-019-1024-5 PMC6441141

[3] Mahmood S Birkaya B Rideout TC Patel MS. Lack of mitochondria-generated acetyl-CoA by pyruvate dehydrogenase complex downregulates gene expression in the hepatic de novo lipogenic pathway. Am J Physiol Endocrinol Metab. 2016;311:E117–27. 27166281 10.1152/ajpendo.00064.2016 PMC4967143

[4] Shiiba I Ito N Oshio H. ER-mitochondria contacts mediate lipid radical transfer via RMDN3/PTPIP51 phosphorylation to reduce mitochondrial oxidative stress. Nat Commun. 2025;16:1508. 39929810 10.1038/s41467-025-56666-4 PMC11811300

[5] Liao WQ Pang Y Yu CA Wen JY Zhang YG Li XH. Novel mutations of mitochondrial DNA associated with type 2 diabetes in Chinese Han population. Tohoku J Exp Med. 2008;215:377–84. 18679013 10.1620/tjem.215.377

[6] Wang S Li R Fettermann A. Maternally inherited essential hypertension is associated with the novel 4263A>G mutation in the mitochondrial tRNAIle gene in a large Han Chinese family. Circ Res. 2011;108:862–70. 21454794 10.1161/CIRCRESAHA.110.231811

[7] Kokaze A Ishikawa M Matsunaga N. Mitochondrial DNA 5178 C/A polymorphism influences the effects of habitual smoking on the risk of dyslipidemia in middle-aged Japanese men. Lipids Health Dis. 2012;11:97. 22857129 10.1186/1476-511X-11-97 PMC3459723

[8] Qin J Han TQ Fei J. [Risk factors of familial gallstone disease: study of 135 pedigrees]. Zhonghua Yi Xue Za Zhi. 2005;85:1966–9. 16313772

[9] Sun D Niu Z Zheng HX. A mitochondrial DNA variant elevates the risk of gallstone disease by altering mitochondrial function. Cell Mol Gastroenterol Hepatol. 2021;11:1211–26.e15. 33279689 10.1016/j.jcmgh.2020.11.015 PMC8053626

[10] Zhu Z Zhang F Hu H. Integration of summary data from GWAS and eQTL studies predicts complex trait gene targets. Nat Genet. 2016;48:481–7. 27019110 10.1038/ng.3538

[11] Fairfield CJ Drake TM Pius R. Genome-wide analysis identifies gallstone-susceptibility loci including genes regulating gastrointestinal motility. Hepatology. 2022;75:1081–94. 34651315 10.1002/hep.32199

[12] McRae AF Marioni RE Shah S. Identification of 55,000 replicated DNA methylation QTL. Sci Rep. 2018;8:17605. 30514905 10.1038/s41598-018-35871-w PMC6279736

[13] Võsa U Claringbould A Westra HJ; BIOS Consortium. Large-scale cis- and trans-eQTL analyses identify thousands of genetic loci and polygenic scores that regulate blood gene expression. Nat Genet. 2021;53:1300–10. 34475573 10.1038/s41588-021-00913-z PMC8432599

[14] Pietzner M Wheeler E Carrasco-Zanini J. Mapping the proteo-genomic convergence of human diseases. Science. 2021;374:eabj1541. 34648354 10.1126/science.abj1541 PMC9904207

[15] Rath S Sharma R Gupta R. MitoCarta3.0: an updated mitochondrial proteome now with sub-organelle localization and pathway annotations. Nucleic Acids Res. 2021;49:D1541–7. 33174596 10.1093/nar/gkaa1011 PMC7778944

[16] Giambartolomei C Vukcevic D Schadt EE. Bayesian test for colocalisation between pairs of genetic association studies using summary statistics. PLoS Genet. 2014;10:e1004383. 24830394 10.1371/journal.pgen.1004383 PMC4022491

[17] Wang Q Dhindsa RS Carss K; AstraZeneca Genomics Initiative. Rare variant contribution to human disease in 281,104 UK Biobank exomes. Nature. 2021;597:527–32. 34375979 10.1038/s41586-021-03855-y PMC8458098

[18] Luo W Brouwer C. Pathview: an R/Bioconductor package for pathway-based data integration and visualization. Bioinformatics. 2013;29:1830–1. 23740750 10.1093/bioinformatics/btt285 PMC3702256

[19] Chen L Zhang YH Lu G Huang T Cai YD. Analysis of cancer-related lncRNAs using gene ontology and KEGG pathways. Artif Intell Med. 2017;76:27–36. 28363286 10.1016/j.artmed.2017.02.001

[20] Szklarczyk D Kirsch R Koutrouli M. The STRING database in 2023: protein-protein association networks and functional enrichment analyses for any sequenced genome of interest. Nucleic Acids Res. 2023;51:D638–46. 36370105 10.1093/nar/gkac1000 PMC9825434

[21] Otasek D Morris JH Bouças J Pico AR Demchak B. Cytoscape automation: empowering workflow-based network analysis. Genome Biol. 2019;20:185. 31477170 10.1186/s13059-019-1758-4 PMC6717989

[22] Warde-Farley D Donaldson SL Comes O. The GeneMANIA prediction server: biological network integration for gene prioritization and predicting gene function. Nucleic Acids Res. 2010;38:W214–20. 20576703 10.1093/nar/gkq537 PMC2896186

[23] Yoo M Shin J Kim J. DSigDB: drug signatures database for gene set analysis. Bioinformatics. 2015;31:3069–71. 25990557 10.1093/bioinformatics/btv313 PMC4668778

[24] Kim S Chen J Cheng T. PubChem in 2021: new data content and improved web interfaces. Nucleic Acids Res. 2021;49:D1388–95. 33151290 10.1093/nar/gkaa971 PMC7778930

[25] Arnold PK Finley LWS. Regulation and function of the mammalian tricarboxylic acid cycle. J Biol Chem. 2023;299:102838. 36581208 10.1016/j.jbc.2022.102838 PMC9871338

[26] Choi I Son H Baek JH. Tricarboxylic acid (TCA) cycle intermediates: regulators of immune responses. Life (Basel). 2021;11:69. 33477822 10.3390/life11010069 PMC7832849

[27] Di Ciaula A Wang DQ Portincasa P. An update on the pathogenesis of cholesterol gallstone disease. Curr Opin Gastroenterol. 2018;34:71–80. 29283909 10.1097/MOG.0000000000000423 PMC8118137

[28] Tsai CJ Leitzmann MF Willett WC Giovannucci EL. Dietary carbohydrates and glycaemic load and the incidence of symptomatic gall stone disease in men. Gut. 2005;54:823–8. 15888792 10.1136/gut.2003.031435 PMC1774557

[29] Xu G Yan T Peng Q. Overexpression of the Lias gene attenuates hepatic steatosis in Leprdb/db mice. J Endocrinol. 2021;248:119–31. 33263565 10.1530/JOE-19-0606

[30] Li T Hasan MN Gu L. Bile acids regulation of cellular stress responses in liver physiology and diseases. eGastroenterology. 2024;2:e100074. 39027418 10.1136/egastro-2024-100074 PMC11257078

[31] Aliyu A Dellschaft N Hoad C. Magnetic resonance imaging reveals novel insights into the dual mode of action of bisacodyl: a randomized, placebo-controlled trial in constipation. Clin Pharmacol Ther. 2025;117:1284–91. 39679695 10.1002/cpt.3532 PMC11993282

[32] Bash LD Turzhitsky V Mark RJ Hofer IS Weingarten TN. Post-operative urinary retention is impacted by neuromuscular block reversal agent choice: a retrospective cohort study in US hospital setting. J Clin Anesth. 2024;93:111344. 38007845 10.1016/j.jclinane.2023.111344

[33] Meng C Ren J Gu H. Association between genetically plasma proteins and osteonecrosis: a proteome-wide Mendelian randomization analysis. Front Genet. 2024;15:1440062. 39119575 10.3389/fgene.2024.1440062 PMC11306153

[34] Lee HY Xu Y Huang Y. The gene for paroxysmal non-kinesigenic dyskinesia encodes an enzyme in a stress response pathway. Hum Mol Genet. 2004;13:3161–70. 15496428 10.1093/hmg/ddh330

[35] Bednarski TK Rahim M Hasenour CM. Pharmacological SERCA activation limits diet-induced steatohepatitis and restores liver metabolic function in mice. J Lipid Res. 2024;65:100558. 38729350 10.1016/j.jlr.2024.100558 PMC11179628

[36] Takayanagi M Sano N Takikawa H. Biliary excretion of olmesartan, an anigotensin II receptor antagonist, in the rat. J Gastroenterol Hepatol. 2005;20:784–8. 15853995 10.1111/j.1440-1746.2005.03792.x

[37] Marzio L. Factors affecting gallbladder motility: drugs. Dig Liver Dis. 2003;35:S17–9. 12974504 10.1016/s1590-8658(03)00088-4

[38] Ma Z Wang X Yin P. Serum metabolome and targeted bile acid profiling reveals potential novel biomarkers for drug-induced liver injury. Medicine (Baltim). 2019;98:e16717. 10.1097/MD.0000000000016717 PMC6708818 31374067

